# Fam20C in Human Diseases: Emerging Biological Functions and Therapeutic Implications

**DOI:** 10.3389/fmolb.2021.790172

**Published:** 2021-12-20

**Authors:** Rongsheng Xu, Huidan Tan, Jiahui Zhang, Zhaoxin Yuan, Qiang Xie, Lan Zhang

**Affiliations:** ^1^ Department of Stomatology, Zigong First People’s Hospital, Zigong, China; ^2^ State Key Laboratory of Biotherapy and Cancer Center, West China Hospital, Sichuan University, Chengdu, China; ^3^ Sichuan Engineering Research Center for Biomimetic Synthesis of Natural Drugs, School of Life Science and Engineering, Southwest Jiaotong University, Chengdu, China

**Keywords:** casein kinase, FAM20c, phosphorylation, diseases, inhibitors

## Abstract

Fam20C, a typical member of Fam20 family, has been well-known as a Golgi casein kinase, which is closely associated with Raine Syndrome (RS). It can phosphorylate many secreted proteins and multiple substrates, and thereby plays a crucial role in biological functions. More importantly, Fam20C has also been found to enhance the metastasis of several types of human cancers, such as breast cancer, indicating that Fam20C may be a promising therapeutic target. Accordingly, some small-molecule inhibitors of Fam20C have been reported in cancer. Taken together, these inspiring findings would shed new light on exploiting Fam20C as a potential therapeutic target and inhibiting Fam20C with small-molecule compounds would provide a clue on discovery of more candidate small-molecule drugs for fighting with human diseases.

## Introduction

Hitherto, more and more proteins have been found to play important physiological functions in various life processes after being phosphorylated. Therefore, protein phosphorylation, as a fundamental regulatory modification of proteins, has gradually become an important research content and has come into people’s sight (Cohen, 2002; Taliabracci et al., 2012; Klement and Medzihradszky, 2017). The first sign of protein kinases’ existence was in 1883, when stoichiometric amounts of phosphate were found in the secreted protein casein. This casein was therefore identified as the first phosphoprotein, but the responsible kinase responsible for it was not clear until 2013 (Tagliabracci et al., 2012). The emergence of a novel atypical protein kinase family, the Fam20 family, solved these problems to a certain extent. These protein kinases are secreted in the Golgi apparatus and seem to be able to phosphorylate many extracellular proteins including casein (Tagliabracci et al., 2013). Protein kinase phosphorylates the substrate through phosphorylation, thus changing the activity of the substrate and mediating most of the signal transduction and cell processes, including transcriptional regulation and metabolic regulation (Manning et al., 2002). Therefore, abnormal protein phosphorylation is the cause of many diseases.

Fam20C, this kinase, and its family members Fam20A and Fam20B define a new family of secretory proteins that collectively regulate a diverse network of secretory pathway components (Palma-Lara et al., 2021). As the most widely studied member of the Fam20 family, Fam20C is a secreted protein with kinase activity. In the process of evolution, Fam20C not only obtained new substrate specificity, but also gained the ability to form evolutionarily conserved homodimers or heterodimers with Fam20A to regulate protein conformation and kinase activity. Although the other two members have a high degree of sequence homology, their biochemical activities are completely different. Fam20B has a unique active site that can recognize Galβ1-4Xylβ1 and is able to regulate the synthesis of proteoglycans by acting as a glycan kinase phosphorylating xylose residues and triggers peptidoglycan biosynthesis. At the same time, all Fam20B subfamily members function as monomers and do not need to form homologous or heterodimers. Fam20A, which did not appear until spinal animals, is a pseudokinase because it lacks active site residues that are essential for kinase activity and binds ATP in a catalytically incapable way, but it also obtains a more optimized ability to form dimers with Fam20C and activate Fam20C (Xiao et al., 2013; Zhang et al., 2018a; Worby et al., 2021).

Fam20C, originally named as Golgi-enriched-fraction casein kinase (GEF-CK) and discovered in 1972, is confirmed by Tagliabracci et al. and Ishikawa et al. as a protein kinase that can phosphorylate the S-x-E/pS (S is serine, x is any one amino acid, E/pS is glutamate or phosphoserine) motif of secreted proteins (Bingham et al., 1972; Tagliabracci et al., 2012). In addition, Fam20C can also recognize amino acid sequences other than the typical S-x-E/pS motif, such as S-x-Q-x-x-D-E-E motif, which indicates that Fam20C-dependent phosphorylation is more extensive than indicated by only those substrates that are phosphorylated within the canonical motif (Brunati et al., 2000). Moreover, FAM20C, expressed in a variety of tissues, has been shown by functional annotation of substrates that it not only is involved in biomineralization but also plays an important role in cell migration and adhesion, wound healing, endopeptidase inhibitor activity, and lipid metabolism disorders (Tagliabracci et al., 2015). Such rich and useful functions have established Fam20C as a major protein kinase. Here, we have summarized the structure and function of Fam20C, discussed its important role in various diseases, and further discussed its potential diagnosis and therapeutic effect. Through an in-depth understanding of these relationships, the biological functions of Fam20C will be further developed, laying a new foundation for studying the role of protein phosphorylation in life processes and diseases.

## Structure and Biological Function of FAM20C

The Fam20C gene is located on chromosome 7, with a full length of 72,410 bp and contains 10 exons (Nalbant et al., 2005). The Fam20C protein sequence includes 584 amino acids, of which the C-terminal contains 350 amino acids that are highly conserved regions and is called conserved C-terminal domain (CCD), and the N-terminal has a signal peptide targeting the secretory pathway, a total of 22 amino acids. Its kinase domain (KD) spans 222 amino acids, from residue 354 to 565 (Cozza and Pinna, 2016). Fam20C is considered an atypical protein kinase and is therefore not included in the “typical” group of human kinases. The kinase core of Fam20C has a two-lobe structure (N-lobe and C-lobe), which is characteristic of all protein kinases. The crystal structure of the *C. elegans* homolog of human Fam20C (ceFam20) displays a large kinase domain and a protein kinase-like fold characterized by being contained in a shell-like structure formed by an N-terminal segment and a novel insertion domain. The N-terminal segment wraps around the lower half of the molecule, forming the base of the C-lobe. The insertion domain has a novel fold and forms a cap-like structure that covers the N-lobe. Therefore, sequence analyses usually failed to identify the Fam20 family as kinases because this domain is hidden in the N-lobe (Xiao et al., 2013). In addition, the Mn/ADP-bound structure is important for nucleotide binding and catalysis as the critical residues. Therefore, the unique architecture of the kinase suggests that Fam20C is an efficient catalyst as opposed to a dynamically regulated enzyme (Cozza and Pinna, 2016). Activation of Fam20C requires the formation of either an evolutionarily conserved homodimer or a heterodimer with Fam20A. Compared with Fam20C itself, Fam20A has a more effective Fam20C binding surface and is a special Fam20C allosteric activator. For example, Ile214A, Ile255A, and Leu365A are unique to Fam20A, and contribute to the formation of an optimized hydrophobic surface for interacting with Fam20C (Zhang et al., 2018a). In a word, the structure of Fam20C is distinct to other “typical” kinases, which makes it more druggable. Therefore, further studying of Fam20c structure can help us understand their biological functions, which can promote the development of more effective new drugs to treat diseases.

**FIGURE 1:**
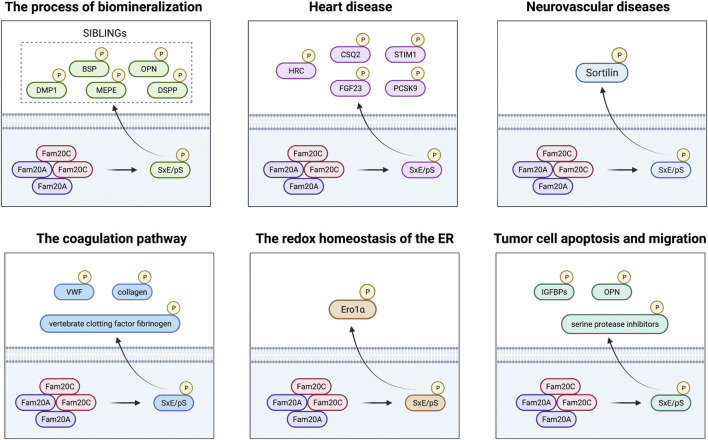
FAM20C plays a role in biomineralization, heart disease, neurovascular disease, blood coagulation, the redox homeostasis of the ER, and tumor disease through different substrates.

Fam20C is not a specific kinase dedicated to phosphorylation of casein, but a ubiquitous protein kinase, which mostly acts on the phosphorylation of many secreted proteins within the SxE/pS motif (Zhou et al., 2009; Salvi et al., 2010). Chromosome 4 harbors a cluster of genes encoding small integrin binding ligand-N-linked glycoproteins (SIBLINGs). The proteins encoded by these genes are involved in binding calcium and are the substrates of Fam20C, mainly five members of the secretory calcium binding phosphoprotein (SCPP) family, namely, dentin matrix protein 1 (DMP1), matrix extracellular phosphoglycoprotein (MEPE), osteopontin (OPN), bone sialoprotein (BSP), and dentin sialophosphoprotein (DSPP), indicating that Fam20C plays an important role in the process of biomineralization (Tagliabracci et al., 2012). Histidine-rich calcium binding protein (HRC) was the first sarcoplasmic reticulum (SR) protein to be discovered as a substrate of Fam20 (Pollak et al., 2017). In addition, other proteins involved in Ca^2+^ signaling include calsequestrin 2 (CSQ2) and matrix interacting molecule 1 (STIM1), as well as fibroblast growth factor 23 (FGF23), which directly causes cardiovascular problems in patients, and proprotein convertase subtilisin 9 (PCSK9), which is related to LDL-cholesterol disorders, which are all key links in the process of Fam20C affecting heart disease (Ezumba et al., 2014; Pollak et al., 2017; Pollak et al., 2018; Ben Djoudi Ouadda et al., 2019). Sortilin is not limited to the expression of a single tissue and is another unique target for Fam20C because of its versatility and its important role in the pathogenesis of a variety of diseases, especially neurovascular diseases. (Xu et al., 2019). Besides, in phosphorylated proteomics screening, the main proteins in plasma and serum such as vertebrate clotting factor fibrinogen, von Willebrand factor (VWF), and collagen were determined as potential substrates for Fam20C. Therefore, the role of Fam20C and its key substrate phosphorylation in the coagulation pathway is also worthy of further study (Tagliabracci et al., 2015; Qiu et al., 2018; Da et al., 2019). Studies have also found that the endoplasmic reticulum (ER) sulfhydryl oxidase ER oxidoreductin 1α (Ero1α), which can regulate the redox homeostasis of the ER, can also be phosphorylated by Fam20C, thereby establishing a new connection between phosphorylation modification and oxidative folding (Zhang et al., 2018b). Finally, many Fam20C phosphorylated substrates are also related to tumor cell apoptosis and migration, including insulin-like growth factor binding proteins (IGFBPs), OPN, and serine protease inhibitors (Rangaswami et al., 2006; Baxter, 2014; Tagliabracci et al., 2015). In short, Fam20C plays an important role in various life processes through phosphorylation of multiple substrates (Figure 1; Supplementary Table S1).

## Diseases Associated With FAM20C

Given the important role of FAM20C in biomineralization and phosphorylating secretory proteins, mutations of FAM20C gene and aberrant function of fam20c kinase are responsible for many diseases, including Raine Syndrome (RS), cancer, and other diseases.

### Raine Syndrome

RS is a bone dysplasia with characteristic features of generalized osteosclerosis, craniofacial anomalies, and intracerebral calcifications (Vishwanath et al., 2014), which is caused by mutations of Fam20C. Hitherto, 42 variants of Fam20C gene have been summarized to be responsible for RS, and they cause either lethal or non-lethal cases (Palma-Lara et al., 2021). For example, mutations of FAM20C gene (MIM *611061) are reported to impair splicing process, leading to the functional loss of proteins and causing disease phenotype (Simpson et al., 2007). More recently, new mutations of Fam20C have been detected to cause lethal RS in two Mexican family siblings, with a duplicated cytosine at position 456 and a deleted cytosine at position 704 in exon 1 (Hernández-Zavala et al., 2020). In addition, whole exome sequencing reveals a non-lethal case of a middle-aged woman with a frameshift insertion c.1107_1108insTACTG (p.Tyr369fs) and a missense substitution c.1375C > G (p.Arg459Gly) in Fam20C gene (Mamedova et al., 2019). Another non-lethal case of RS happens in a Brazil patient carrying a homozygous missense variant c.1487C > T at exon 9 of FAM20C (NM_020223.4), who displays mild facial dysmorphia, without hypoplastic nose, micrognathia, low set ears, or depressed nasal bridge (Ferreira et al., 2021).

FGF23-related hypophosphatemia is reported in non-lethal RS patients and is found to be associated with FAM20C mutations (Simpson et al., 2009; Rafaelsen et al., 2013). Given the causative role of mutant Fam20C gene in RS pathogenesis, some researchers have investigated detailed pathogenetic mechanism of mutant Fam20C in preclinical experiments (Cozza and Pinna, 2016). Six mutant Fam20C proteins (T268M, P328S, R408W, D451N, D478A, and R549W) responsible for RS were demonstrated to cause impaired kinase activity and hinder DMP1 transcription, ultimately leading to FGP-related hypophosphatemia in rat osteosarcoma UMR-106 cells (Kinoshita et al., 2014). Recently, mice that were knocked out of Fam20C showed vertebral abnormalities and decreased β-catenin, indicating that Fam20C loss of function may affect vertebral development through modulating Wnt/β-catenin pathway (Huang et al., 2021).

### Cancer

Recently, the role of Fam20C in tumorigenesis has been illuminated and widely reported, making it a possible biomarker and potentially therapeutic target for diverse cancers (Simpson et al., 2009).

Its association with triple-negative breast cancer (TNBC) was first reported in 2015 (Tagliabracci et al., 2015), as the phosphorylation of a large number of its substrates was identified to play a role in adhesion and migration of MDA-MB-231 cells and U2OS cells, such as IGFBP7 and cadherin-2 (CDH2). Fam20C knockout (KO) MDA-MB-231 cells were demonstrated with less malignant invasion and the phosphorylation of its substrate IGFBP7 was responsible for the changes. In a later study, Fam20C knockdown with siRNA also impaired the metastasis of MDA-MB-231 cells while mildly affecting cell proliferation, indicating the possible oncogenic effect of Fam20C in TNBC (Zhao et al., 2021). More recently, a genome-wide analysis uncovered that high expression and hypomethylation of Fam20C were correlated with poor prognosis in hypoxia-related lung adenocarcinoma (LUAD), in which hypoxia condition promoted FAM20C gene expression, making Fam20C a potential biomarker of LUAD (Li et al., 2020). Subsequently, a study has revealed the expression patterns of Fam20C in pan-cancer, of which increased expressions were correlated with poor prognosis in bladder urothelial carcinoma (BLCA), brain lower grade glioma (LGG), and stomach adenocarcinoma (STAD), making Fam20C a potential prognostic biomarker in those tumors. Particularly, high expression of FAM20C was found to affect lymphatic metastasis of STAD (Liu et al., 2021). The altered infiltration levels of immune cells, including B cells, CD8+T cells, CD4+T cells, macrophages, neutrophils, and dendritic cells were considered to be responsible for its correlation with cancers, while the expression of FAM20C also assisted the polarization of tumor-associated macrophages (TAM), activation of Treg cells and T helper cells, and induction of T-cell exhaustion (Liu et al., 2021). Moreover, augmented Fam20C expression was found to be positively correlated with the malignancy of gliomas, which makes the expression of Fam20C a possibly diagnostic marker of malignant gliomas. Also, patients with higher Fam20C expression were more resistant to radiotherapy and chemotherapy. In line with the bioinformatics analysis, Fam20C knockdown significantly impaired the invasion and migration of human glioblastoma LN229 cells (Du et al., 2020). More importantly, a wide range of secreted proteins phosphorylated by Fam20C were found to be associated with different cancers (Zhao et al., 2021). Fibronectin 1 (FN1) was identified to interact with Fam20C and promote tumor cell migration (Du et al., 2020). OPN, a substrate of Fam20C involved in cell differentiation (Zhao et al., 2018), was overexpressed in bladder cancer tissues and OPN knockdown could suppress proliferation and invasion of human bladder cancer T24 cell *via* the JAK1/STAT1 pathway (Zhang et al., 2020). In addition, myeloid Fam20C phosphorylates OPN and decreases its secretion, thereby regulating osteoclast differentiation and decelerating breast cancer bone metastasis *in vivo* (Zuo et al., 2021). Another substrate, BMP4, is also involved in breast cancer bone metastasis, in which Fam20C facilitates the growth of human breast cancer MDA-BoM-1833 cells and its bone metastasis through phosphorylation of BMP4 to induce osteoclast differentiation (Zuo et al., 2021). IGFBPs 3 and 7, which are both substrates of Fam20C (Liu et al., 2018), were reported to enhance the metastasis of colorectal cancer (CRC) sW480 and Caco2 cells (Georges et al., 2011; Supplementary Table S2).

### Other Human Diseases

Besides IGFBP7 and CDH2, which play an important role in TNBC metastasis, the comprehensive article in 2015 identified many other substrates of Fam20C that were involved in other diseases, including Fetuin A (FetA) and histidine-rich calcium binding protein (HRC) (Tagliabracci et al., 2015). Phosphorylated FetA was reported to play a part in insulin resistance in type 2 diabetes mellitus (T2DM) through binding the β-subunit of the insulin receptor and hindering insulin signaling (Ochieng et al., 2018). A study showed that the phosphorylation of FetA at Ser 312 is time-dependent on Fam20C, whereas the biological function of FAM20C-dependent FetA phosphorylation remains unknown (Kovářová et al., 2021). Another study originally revealed the possible regulatory effects of Fam20C on (pro)insulin production and correlative secretory pathway trafficking, establishing connections between Fam20C function and the development of diabetes (Kang et al., 2019). HRC is a 170-kDa protein with high capacity for Ca^2+^ binding and is critical in the modulation of cardiomyocyte SR Ca^2+^ homeostasis (Arvanitis et al., 2018). Recently, the relationship between Fam20C and SR Ca^+^ handling machinery has been illuminated, demonstrating that the phosphorylation of HRC at Ser96 by Fam20C regulates the interactions of HRC and triadin and sarco/endoplasmic reticulum Ca2^+^ adenosine triphosphatase-2a (SERCA2a), maintains the SR Ca^+^ homeostasis, and finally protects patients from cardiac arrhythmias (Pollak et al., 2017). A follow-up study identified Stim1 and CSQ2 as new substrates of Fam20C, which are important in controlling the SR Ca2^+^homeostasis. The loss of Fam20C-dependent phosphorylation of these two proteins may underlie the development of heart failure in Fam20C KO mice (Pollak et al., 2018). In addition, the possible pathogenesis of Fam20C in coronary artery disease has been revealed, of which the Fam20C-dependent phosphorylation of sortlin at Ser825 is integral in the process of smooth muscle cell (SMC) calcification (Goettsch et al., 2016). One latest study reported the decreased expression of Fam20C in behavioral variant frontotemporal dementia (bvFTD) with autoimmune disease (Bright et al., 2021).

## Therapeutic Potential for Targeting FAM20C

**FIGURE 2:**
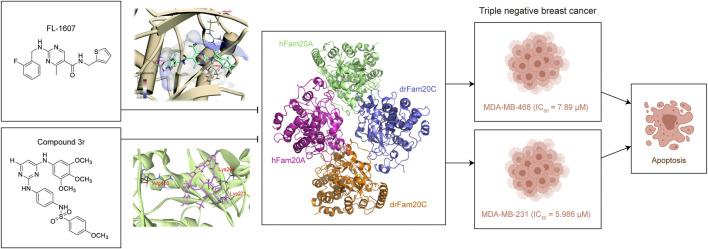
Fam20C inhibitors are designed to treat breast cancer. There are two inhibitors of Fam20C, FL-1607 (Qin et al., 2016) and compound 3r (Zhao et al., 2021).

Considering the important role of Fam20C in various diseases, especially its role in tumorigenesis, targeting Fam20C is a potential treatment for diseases including cancer in the future (Wu et al., 2021). Some attempts to design inhibitors of Fam20C for treating cancer, focused in TNBC, have emerged. For the first time, FL-1607 was identified through virtual screening and interacted well with Fam20C, with better stability (RSMD reached its dynamic equilibrium after 2 ns simulation) and compactness (RG of 1.462 ± 0.0073 nm), as well as lower flexibility (RMSF of 0.0805 ± 0.0353 nm; mean ± SD) when binding with Fam20C. Meanwhile, it showed good antiproliferative property in human breast cancer MCF-7, MDA-MB-231 and MDA-MB-468 cells, with the best IC50 of 7.89 μM in MDA-MB-468 cells. Moreover, FL-1607 was shown to induce apoptosis and suppress migration of MDA-MB-468 cells. The researchers found that Fam20C harbored unique amino acids residues in ATP-binding sites, which makes Fam20C more possible to be selectively target by small molecules (Qin et al., 2016). Encouraged by this study and the x-ray crystal structure of Fam20C (Zhang et al., 2018a), a new inhibitor of Fam20C was identified in 2021 (Zhao et al., 2021). Optimized from F2078-0064, compound **3r** showed good kinase inhibitory ability against Fam20C (6.243 μM), with IC50 of 5.986 μM in MDA-MB-231 cells. **3r** killed cancer cells through apoptosis induction and could significantly block migration of MDA-MB-231 cells. In addition, **3r** inhibited tumor growth in MDA-MB-231 xenograft mouse model through inducing apoptosis (Zhao et al., 2021) (Figure 2).

Two inhibitors of Fam20C mentioned above shed light on the therapeutic potential for targeting Fam20C in cancer, providing successful examples for future discovery of new Fam20C inhibitors. However, in-depth investigations of therapies targeting Fam20C remain limited, and no studies illuminated its therapeutic effects in heart diseases and diabetes, in which experimental evidence confirmed the important pathogenetic role of Fam20C. The two existing inhibitors may be good tools for studying biological and pathogenetic functions of Fam20C while their application for treating other diseases associated with Fam20C warrants further investigations. Additionally, the wide distribution of Fam20C in bone, heart, kidney, etc. may make therapeutics targeting Fam20C easily cause side effects in unexpected organs or tissues, thus requiring more comprehensive understanding of Fam20C functions in different tissues and calling for precise delivery of therapeutics. Moreover, molecular subtyping of diseases, especially of cancers with aberrant expression of Fam20C, represents a future direction.

## Conclusion

Of note, the Fam20 family is composed of three secreted proteins, including Fam20A, Fam20B, and Fam20C; among them, Fam20C could phosphorylate a series of downstream secreted proteins. Fam20C is a typical member of Fam20 family, which has been well-known as a Golgi casein kinase that can be identified during hematopoietic differentiation (Nalbant et al., 2005). Interestingly, the other two members Fam20A and Fam20B have a high sequence homology with Fam20C, but their biological activities are completely different. Thus, Fam20C has its unique biological function in several cellular processes (Worby et al., 2021).

Recently, Fam20C, which is closely associated with RS, has been reported to phosphorylate more than 100 types of secreted proteins and more multiple substrates, thereby playing a key role in regulation of their complicated biological function (Tagliabracci et al., 2015). Accordingly, accumulating evidence has been demonstrating that Fam20C has several links to many types of human diseases, such as RS, cancer, and others. More importantly, Fam20C has also been found to enhance the metastasis of several types of human cancers, such as breast cancer and CRC, indicating that Fam20C may be a promising therapeutic target for the current drug development. More recently, some small-molecule inhibitors of Fam20C (e.g., FL-1607 and 3r) have been reported to induce apoptosis as well as to prevent metastasis in breast cancer, which may reveal Fam20C as a druggable target for cancer therapy (Qin et al., 2016; Zhao et al., 2021).

With the rapid development of structure biology and artificial intelligence (AI), the tertiary structure of Fam20C and even the quaternary structure of Fam20C-Fam20A have been elucidated (Zhang et al., 2018a). Therefore, the structure-guided design of Fam20C inhibitors will provide more precisely small-molecule inhibitors for potential therapy. Also, some AI technologies, such as Alphafold2 would also provide more potential selectively small-molecule inhibitors for possible pharmaceutical applications. Especially when the experimental structure is not available, Alphafold2 may provide more accurate model for virtual screening (Cramer, 2021). In a nutshell, these inspiring findings would shed new light on exploiting Fam20C as a potential therapeutic target and inhibiting Fam20C with small-molecule compounds will provide a new clue on discovery of more candidate drugs in the treatment of human diseases in the future.

## Glossary

AI: artificial intelligence
BLCA: bladder urothelial carcinoma
BSP: bone sialoprotein
bvFTD: behavioral variant frontotemporal dementia
CRC: colorectal cancer
CDH2: cadherin-2
CSQ2: calsequestrin 2
DMP1: dentin matrix protein 1
DSPP: dentin sialophosphoprotein
ER: endoplasmic reticulum
Ero1α: ER oxidoreductin 1α
FetA: Fetuin A
FGF23: fibroblast growth factor 23
FN1: fibronectin 1
GEF-CK: Golgi-enriched-fraction casein kinase
HRC: histidine-rich calcium binding protein
IGFBP: insulin-like growth factor binding protein
KD: kinase domain
KO: knockout
LGG: brain lower grade glioma
LUAD: lung adenocarcinoma
MEPE: matrix extracellular phosphoglycoprotein
OPN: osteopontin
PCSK9: proprotein convertase subtilisin 9
RS: Raine syndrome
SCPP: secretory calcium binding phosphoprotein
SERCA2a: sarco/endoplasmic reticulum Ca^2+^ adenosine triphosphatase-2a
SIBLING: small integrin binding ligand-N-linked glycoprotein
SMC: smooth muscle cell
SR: sarcoplasmic reticulum
STAD: stomach adenocarcinoma
STIM1: matrix interacting molecule 1
T2DM: type 2 diabetes mellitus
TAM: tumor-associated macrophages
TNBC: triple-negative breast cancer
VWF: von Willebrand factor
