# Supplementary material for: Fam20C in Human Diseases: Emerging Biological Functions and Therapeutic Implications
Source: Front Mol Biosci. 2021 Dec 20;8:790172. doi: 10.3389/fmolb.2021.790172 (PMC8721277; doi:10.3389/fmolb.2021.790172)
Supplement: Supplementary file 1 [file DataSheet1.docx]

**Table S1. Fam20C substrates and related biological processes**

| **Substrate** | **Protein name** | **Biological process** | **Ref.** |
| --- | --- | --- | --- |
| DMP1 | Dentin matrix protein 1 | Biomineralization. | (Tagliabracci et al., 2012) |
| MEPE | Matrix extracellular phosphoglycoprotein | Biomineralization. | (Tagliabracci et al., 2012) |
| OPN | Osteopontin | Biomineralization. | (Tagliabracci et al., 2012) |
| BSP | Bone sialoprotein | Biomineralization. | (Tagliabracci et al., 2012) |
| DSPP | Dentin sialophosphoprotein | Biomineralization. | (Tagliabracci et al., 2012) |
| HRC | Histidine-rich calcium binding protein | Heart disease; participating in Ca2+ signaling. | (Pollak et al., 2017) |
| CSQ2 | Calsequestrin 2 | Heart disease; participating in Ca2+ signaling. | (Ezumba et al., 2014;Pollak et al., 2017;Pollak et al., 2018;Ben Djoudi Ouadda et al., 2019) |
| STIM1 | Stromal interaction molecule 1 | Heart disease; participating in Ca2+ signaling. | (Ezumba et al., 2014;Pollak et al., 2017;Pollak et al., 2018;Ben Djoudi Ouadda et al., 2019) |
| FGF23 | Fibroblast growth factor 23 | Heart disease; causing cardiovascular problems. | (Ezumba et al., 2014;Pollak et al., 2017;Pollak et al., 2018;Ben Djoudi Ouadda et al., 2019) |
| PCSK9 | Proprotein convertase subtilisin 9 | Heart disease; LDL-cholesterol disorders. | (Ezumba et al., 2014;Pollak et al., 2017;Pollak et al., 2018;Ben Djoudi Ouadda et al., 2019) |
| SORT1 | Sortilin | Neurovascular diseases | (Xu et al., 2019) |
| Vertebrate clotting factor fibrinogen | / | The coagulation pathway. | (Tagliabracci et al., 2015;Da et al., 2019) |
| VWF | Von Willebrand factor | The coagulation pathway. | (Tagliabracci et al., 2015;Da et al., 2019) |
| Collagen | / | The coagulation pathway. | (Qiu et al., 2018) |
| Ero1α | ER oxidoreductin 1α | The redox homeostasis of the endoplasmic reticulum. | (Zhang et al., 2018) |
| IGFBPs | Insulin-like growth factor binding proteins | Tumor cell apoptosis and migration. | (Rangaswami et al., 2006;Baxter, 2014;Tagliabracci et al., 2015) |
| OPN | Osteopontin | Tumor cell apoptosis and migration. | (Rangaswami et al., 2006;Baxter, 2014;Tagliabracci et al., 2015) |
| Serine protease inhibitors | / | Tumor cell apoptosis and migration. | (Rangaswami et al., 2006;Baxter, 2014;Tagliabracci et al., 2015) |

**Table S2. The role of Fam20C in the different types of tumors**

| **Cancer type** | **Cell type** | **Condition of Fam20** | **Relevant Fam20C substrates** | **Ref.** |
| --- | --- | --- | --- | --- |
| Triple negative breast cancer | MDA-MB-231; MDA-BoM-1833 | \ | IGFBP7 and CDH2; BMP4 | (Tagliabracci et al., 2015;Zuo et al., 2021) |
| Lung adenocarcinoma | \ | overexpressed | \ | (Li et al., 2020) |
| Bladder urothelial carcinoma | \ | overexpressed | \ | (Liu et al., 2021) |
| Lower grade glioma | \ | overexpressed | \ | (Liu et al., 2021) |
| Stomach adenocarcinoma | \ | overexpressed | \ | (Liu et al., 2021) |
| Gliomas | LN229 | overexpressed | FN1 | (Du et al., 2020) |
| Bladder cancer | T24 | \ | OPN | (Zhao et al., 2018) |
| Colorectal cancer | sW480; Caco2 | \ | IGFBPs 3 and 7, | (Georges et al., 2011) |
